# Piver Type II vs. Type III Hysterectomy in the Treatment of Early-Stage Cervical Cancer: Midterm Follow-up Results of a Randomized Controlled Trial

**DOI:** 10.3389/fonc.2018.00568

**Published:** 2018-11-28

**Authors:** Hengzi Sun, Dongyan Cao, Keng Shen, Jiaxin Yang, Yang Xiang, Fengzhi Feng, Lingying Wu, Zhenyu Zhang, Bin Ling, Lei Song

**Affiliations:** ^1^Department of Obstetrics and Gynecology, Peking Union Medical College Hospital, Chinese Academy of Medical Sciences and Peking Union Medical College, Beijing, China; ^2^Department of Gynecological Oncology, Cancer Hospital Chinese Academy of Medical Sciences, Beijing, China; ^3^Department of Obstetrics and Gynecology, Beijing Chao-Yang Hospital, Beijing, China; ^4^Department of Obstetrics and Gynecology, China-Japan Friendship Hospital, Beijing, China; ^5^Department of Obstetrics and Gynecology, Chinese PLA General Hospital, Beijing, China

**Keywords:** cervical cancer, modified radical hysterectomy, early stage, quality of life, security

## Abstract

**Introduction:** With the expansion of value-based medicine, we explore whether using type III hysterectomy to treat low-risk, early-stage cervical cancer constitutes overtreatment. In present study, we evaluate the midterm safety and postoperative quality of life of patients who underwent type II hysterectomy vs. type III hysterectomy with systematic lymphadenectomy for low-risk early-stage cervical cancer (International Federation of Gynecology and Obstetrics (FIGO) IA2-IB1; maximum tumor diameter < 2 cm).

**Patients and methods:** The main study was a multicenter, phase III, randomized controlled trial (NCT02368574, https://www.clinicaltrials.gov/ct2/show/NCT02368574). Patients meeting the criteria were randomly divided into type II and type III hysterectomy groups between 2015 and 2018. Midterm outcomes were analyzed at 36 months after the first eligible patient was enrolled. The primary end point was disease-free survival, and the secondary end point was postoperative quality of life.

**Results:** A total of 97 patients were preliminarily enrolled, 93 of whom were included in the final analysis. The general information of the two groups did not differ. The 2-year DFS rate in the type II group was 100% compared with 97.9% in the type III group (*P* > 0.05). Compared to the type III group, the patients who underwent type II hysterectomy showed a shorter surgical time (163 ± 18.8 min vs. 226 ± 16.4 min, *P* = 0.014), less intraoperative blood loss (174 ± 27.7 ml vs. 268 ± 37.4 ml, *P* = 0.047), less postoperative urinary retention (5/46 vs. 11/47 cases, *P* = 0.109), and milder bladder injuries. The postoperative symptom experience scores of the type II group were significantly lower than those of the type III group. Moreover, the postoperative sexual/vaginal functioning and lubrication scores of the type II group were significantly lower than those of the type III group in subgroup analyses of patients who did not undergo postoperative chemoradiotherapy. Sexual apprehension scores were increased postoperatively in both groups.

**Conclusion:** Based on the midterm analysis, the two groups show considerable security within 2 years after surgery, but long-term security requires further analysis. Type II hysterectomy can effectively reduce the surgical time and intraoperative blood loss, decrease postoperative complications, and improve the quality of life of early-stage cervical cancer patients.

## Introduction

Cervical cancer is the most common malignant gynecologic tumor and severely affects the mental and physical health of women. Although effective screening has significantly reduced the incidence of advanced cervical cancer in the last 20 years, the incidence of early-stage cervical cancer in young women shows an increasing trend (1). The standard surgical method for stage IA2-IB1 cervical cancer is radical (type III) hysterectomy with systematic lymphadenectomy. The 24th International Federation of Gynecology and Obstetrics (FIGO) conference reported that the 5-year survival rates of stage IA2 and IB1 cervical squamous cell carcinoma patients were 99.1 and 92.3%, respectively. In addition, these rates among adenocarcinoma patients were 97.1 and 94.2%, respectively (2).

The Piver–Rutledge–Smith classification published in 1974 which includes the class I–V category, has gained substantial popularity (3). Piver class I hysterectomy aims to ensure removal of all cervical tissue. Piver class II hysterectomy and Piver class III hysterectomy are modified radical hysterectomy and radical hysterectomy procedures, respectively. Piver class IV hysterectomy aims to completely remove all periureteral tissue, with more extensive excision of the perivaginal tissues and excision of the internal iliac vessels along this part of the pelvic wall. The objective of Piver class V hysterectomy is to remove central recurrent cancer involving portions of the distal ureter or bladder, although this procedure is no longer used. Recently, many updated versions of hysterectomy based on the Piver–Rutledge–Smith classification have been proposed. The advanced Querleu and Morrow surgical classification system, which was proposed in 2008, represents an updated version of the Piver classification (4) and describes four types of radical hysterectomy (types A, B, C, and D), with a few additional subtypes, such as subtype C1, which includes nerve preservation, and subtype C2, which does not include preservation of autonomic nerves. Type B corresponds to Piver class II hysterectomy, or modified radical hysterectomy, and type C corresponds to Piver class III hysterectomy, or radical hysterectomy. The advanced National Comprehensive Cancer Network (NCCN) guidelines recommend type B and type C hysterectomy as the surgical methods for cervical cancer patients with stage IA2 and IB1 disease. However, to distinguish patients requiring subtype C1 hysterectomy, the Piver classification was used in the present study. Standard radical hysterectomy (Piver type III hysterectomy) requires resection of parametrial tissues close to the pelvic wall and the upper 1/3 or 1/2 of the vagina to ensure negative margins and surgical thoroughness (5). Because the surgical range is large, various intraoperative and postoperative complications occur frequently, such as bleeding, other organ injuries, urinary retention, and dyspareunia. These complications substantially compromise postoperative sexual, bladder, and physiological functions of patients and severely affect their quality of life (6, 7). However, in clinical practice, the risks of parametrial tissue infiltration and lymph node (LN) metastasis in low-risk, early-stage cervical cancer patients (FIGO stage: IA2-IB1; maximum tumor diameter < 2 cm) are very low (2, 8, 9).

With changes in medical models and the expansion of value-based medicine, using traditional Piver type III hysterectomy as the standard treatment method for low-risk, early-stage cervical cancer has been challenged. Does the use of type III hysterectomy to treat low-risk, early-stage cervical cancer constitute overtreatment (6)? Can modified radical hysterectomy (Piver type II hysterectomy) with a reduced surgical range be used to minimize postoperative complications and improve the quality of life of patients? No clinical studies have provided strong medical evidence to answer these questions. Therefore, we comprehensively, systematically, and scientifically evaluated the clinical value of type II/type III hysterectomy with systematic lymphadenectomy to identify an appropriate surgical method for low-risk, early-stage cervical cancer patients.

Recently, the Laparoscopic Approach to Cervical Cancer (LACC) study reported that laparoscopic or robot-assisted radical hysterectomy was associated with lower rates of disease-free survival and overall survival than open abdominal radical hysterectomy among women with early-stage cervical cancer (10). However, the prospective study lacks some relevant data, such as tumor size in 1/3 of the cases and information regarding paraventricular and vaginal involvement in 7–10% of the cases, and only 39.5% of the cases reached the 4.5-year follow-up end point. In addition, the 2019 NCCN guidelines, version 2, suggest that laparotomy, laparoscopy, or robotic laparoscopy is an acceptable radical hysterectomy approach, and laparoscopic radical hysterectomy has been demonstrated to be associated with more favorable morbidity profiles, lower costs of care, and comparable survival relative to abdominal radical hysterectomy through decades of research (11–14). Therefore, Piver II hysterectomy and Piver III hysterectomy were performed through laparoscopy in the initial of present study.

## Patients and methods

### General information

Patients diagnosed with cervical cancer (FIGO stage: IA2 and IB1, maximum tumor diameter < 2 cm) in five research centers were enrolled in a multicenter, phase III, randomized controlled trial (ClinicalTrials.gov identifier: NCT02368574) between March 2015 and March 2018. The estimated number of enrolled participants for the final analysis was as least 180 with a noninferiority margin of 10%, an alpha error of 0.05, and a power for 0.8. Significance different survival time between two groups is defined as the early endpoint. The patients were randomly divided into type II and type III hysterectomy groups by the Interactive Web Response System (IWRS). The five research centers include: Peking Union Medical College Hospital, Cancer Hospital Chinese Academy of Medical Sciences, Beijing Chao-Yang Hospital, Chinese PLA General Hospital, and China-Japan Friendship Hospital.

### Inclusion criteria

(1) 18–60 years old; (2) FIGO stage IA2–IB1; (3) MRI examination showing a maximum tumor diameter < 2 cm and a depth of interstitial infiltrates < 50%; (4) Histological diagnosis of squamous cell carcinoma, adenocarcinoma, or adenosquamous carcinoma; and (5) Signed informed consent.

### Exclusion criteria

(1) High-risk histological types; (2) CT or MRI evaluation indicative of LN-positive disease; (3) A history of neoadjuvant chemotherapy; (4) Pregnancy; (5) Strong desire to retain fertility; (6) Surgical contraindications; (7) Previous history of intestinal obstruction, recurrent pelvic inflammatory disease, pelvic tuberculosis, or pelvic or abdominal tumor surgery; (8) Previous history of bladder or ureteral surgery, previous urinary retention, urinary incontinence or fecal incontinence; and (9) No written informed consent. All patients underwent MRI or contrast-enhanced CT, and the depth of interstitial infiltrates was reviewed by two radiologists at least in the study group until the patients could be enrolled. Pathology and radiology were reviewed in each individual center, and the MRI or CT data will be saved for regular centralized sampling inspection.

### Surgical methods

#### Piver type II hysterectomy

Piver type II hysterectomy (3) is also known as modified radical hysterectomy, and the corresponding surgical range is wider than that of type I extrafascial total hysterectomy, with resection of more parametrial tissues and preservation of the blood supply to the distal ureter and bladder. Separation of the ureter started from the ureteral tunnel, the entire vesicouterine ligament was preserved, and 1/2 of the uterosacral ligament and 1–2 cm of the vagina were resected. In addition, the pelvic LNs were dissected. Type II total hysterectomy was performed laparoscopically (Supplementary Figure 1A).

#### Piver type III hysterectomy

Piver type III hysterectomy (3), which is also known as radical hysterectomy, is the traditional surgical method for the treatment of early-stage cervical cancer. This extended hysterectomy technique requires opening the lateral bladder fossa and lateral rectal fossa. The uterine artery was ligated at the beginning of the internal iliac artery, the ureteral tunnel was completely freed, and the ureter was pushed down to the ureterovesical junction. Next, all ligaments and connective tissues joining the anterior and posterior sides and the bilateral sides of the uterus were separated and resected. The uterosacral ligament was resected close to the sacrum, and the cardinal ligament was resected close to the pelvic lateral wall. After the paravaginal connective tissues were all resected, the top 1/3 or 1/2 of the vagina was resected, and the resection margin was 3–4 cm from the cervical lesion. In addition, the pelvic LNs were dissected. Type III hysterectomy was performed through a laparoscope (Supplementary Figure 1B).

#### Quality assurance of surgery

According to the Standard Operating Procedures (SOPs) of our study, all surgical procedures were videotaped and postoperative specimens with scale plate were photographed and uploaded to our EDC system (Supplementary Figure 1). A quality-control team consisted of five gynecological oncologists was established to evaluate the surgical procedures.

### Observation indicators

The basic information of the patients was statistically analyzed. Eligibility screening, quality of life assessments, and urodynamic examinations were completed 6 weeks before surgery. Intraoperative indicators included the surgical time, intraoperative blood loss, and other organ and blood vessel injuries. Perioperative indicators included intestinal obstruction, infection, thromboembolic diseases, and urinary retention. The postoperative follow-up included a regular follow-up, urodynamic examinations at 6 months postoperatively, and quality of life assessments at 6, 12, and 24 months postoperatively using the Chinese version of the European Organization for Research and Treatment of Cancer (EORTC) Quality of Life Questionnaire—Cervical Cancer Module (QLQ-CX24), the Female Sexual Distress Scale (FSDS), and the Female Sexual Functioning Index (FSFI) (15–17). These questionnaires have been tested and corrected in Asian countries (including China).

### Statistical analysis

Quantitative data were compared by a *t*-test or the Wilcoxon rank sum test. Qualitative data were analyzed using the chi-square test or Fisher's exact test. Survival was calculated using the Kaplan-Meier method. ANOVA with a *post hoc* test and Fisher's exact test were also used for comparisons among groups. Midterm results were analyzed 36 months after the first eligible patient was enrolled. The primary end point was disease-free survival (DFS), and the secondary end point was postoperative quality of life. Patients with positive LNs intraoperatively or on frozen section received standard treatment according to the advanced NCCN guidelines and were included in the final statistical analysis as a subgroup of the experimental group according to SOPs.

## Results

**Figure 1:**
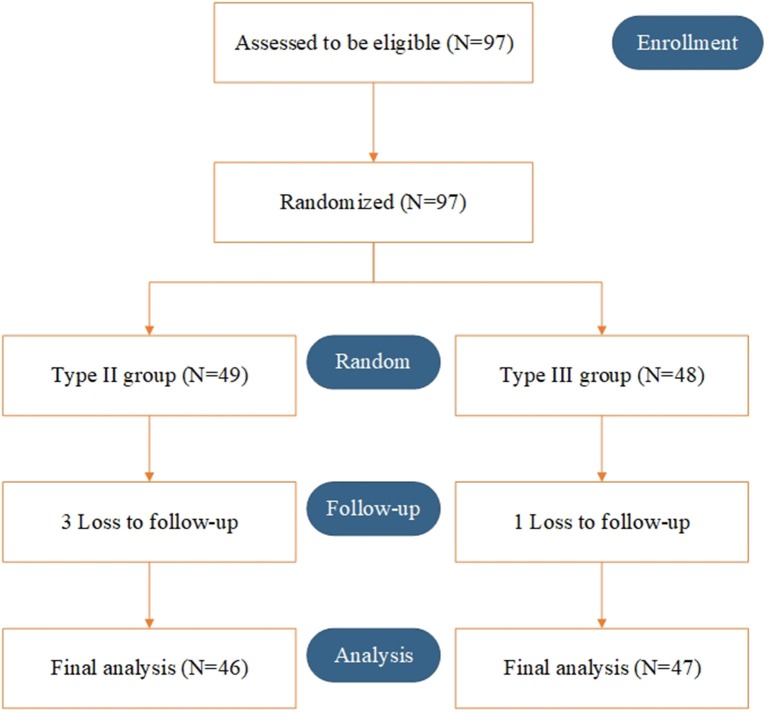
Consort diagram.

Between March 2015 and March 2018, a total of 97 patients were randomly enrolled. Overall, 49 and 48 patients underwent type II and type III hysterectomy with systematic lymphadenectomy, respectively. Three patients in the type II group and 1 patient in the type III group were lost to follow-up. Ninety-three patients (46 in the type II group and 47 in the type III group) were included in the final analysis (Figure 1).

The general information of the patients is shown in Table 1. Age, gravidity and parity history, body mass index (BMI), tumor stage, menstrual status, sexual life, marital status, working condition, education level, and hypogastric surgical history (including previous surgery related to intestinal obstruction, recurrent pelvic inflammatory disease, pelvic tuberculosis, pelvic, and abdominal tumors or the bladder/ureter) were not significantly different between the two groups (*P* > 0.05).

**Table 1:** Clinical and demographic characteristics of the study participants.

**Characteristic**	**Type II cohort *N* = 46**	**Type III cohort *N* = 47**	***P*-value**
Median age	45(31–60)	46(35–60)	NS
Parity	1.4	1.6	NS
BMI	25.7	26.5	NS
Prior intra-abdominal surgery (%)	4(8.7)	3(6.4)	NS
**Pathological type**			
Squamous	44	41	NS
Adenosquamous	2	6
**Figo stage**			
IA2	1	1	NS
IB1	45	46
**Menopausal status (%)**			
Pre-menopausal	35 (76.1)	32 (67.4)	NS
Post-menopausal	11 (23.9)	15 (33.6)
**Sexually active (%)**			
Yes	35 (76.1)	31 (66.0)	NS
NO	11 (23.9)	16 (34.0)
**Partner status (%)**			
Partnered	43 (93.5)	41 (87.2)	NS
Non-partnered/single	3 (6.5)	6 (12.8)
**Employment (%)**			
Yes	32 (69.6)	34 (72.3)	NS
NO	14 (30.4)	13 (27.7)
**Educational level (%)**			
Post-secondary	34 (73.9)	36 (76.6)	NS
Other	12 (26.1)	11 (23.4)

*BMI, body mass index; FIGO, international federation of gynecology and obstetrics; NS, not significant*.

The comparison of surgery-related information between the two groups is shown in Table 2. The results show that the hospitalization time did not differ between the two groups. However, compared to the type III group, the surgical time was significantly shorter (163 ± 18.8 min vs. 226 ± 16.4 min, *P* = 0.014) and the intraoperative blood loss was significantly less (174 ± 27.7 ml vs. 268 ± 37.4 ml, *P* = 0.047) in the type II group. A total of three patients experienced intraoperative complications, including two patients with intraoperative large blood vessel injuries (one in each group) and one patient with a ureteral injury (type II). In the type II group, 1 case of parametrial infiltration and 1 case of pelvic LN metastasis combined with myometrial invasion were noted. Four cases of pelvic LN metastasis were noted in the type III group, 2 of which were accompanied by myometrial infiltration (*P* = 0.413). These six patients subsequently received postoperative radiotherapy and chemotherapy. The mean postoperative first aerofluxus times in the type II and type III groups were 1.8 and 2 days (*P* = 0.803), and the mean times to the first bowel movement were 3.3 and 3.6 days, respectively (*P* = 0.841). Five patients had perioperative complications, including four patients with infection (one patient in the type II group and three patients in the type III group) and one patient with a thromboembolic event (the type II group). Urinary retention within 14 days postoperatively occurred in 5/46 and 11/47 cases in the type II and type III hysterectomy groups, respectively (*P* = 0.109). Twenty-two of 93 (23.7%) patients had lymphovascular space invasion (LVSI) after surgery and received postoperative radiotherapy and chemotherapy, 10 of whom were in the type II group and 12 of whom were in the type III group (*P* = 0.667). The rates of intraoperative complications, parametrial infiltration, LVSI, pelvic LN metastasis, and myometrial infiltration were not significantly different between the two groups.

**Table 2:** Comparison of surgical characteristics in patients treated with Type II and Type III hysterectomy cohort.

	**Type II cohort *N* = 46**	**Type III cohort *N* = 47**	***P*-value**
Length of hospital stay (days)	10.4 ± 1.22	9.25 ± 1.42	NS
Operating time (min)	163 ± 18.8	226 ± 16.4	0.014
Blood loss (ml)	174 ± 27.7	268 ± 37.4	0.047
Transfusion [ml (n)]	200 (1)	510 (4)	NS
Intraoperative complications	2	1	NS
Perioperative complications	2	3	NS
Lymphatic metastasis/beyond perimetrium	2	4	NS
Postoperative first aerofluxus time (days)	1.8 ± 0.65	2 ± 0.47	NS
Postoperative first bowel movement (days)	3.3 ± 1.21	3.6 ± 0.88	NS
Urine retention	5	11	0.109
LVSI	10	12	NS

*LVSI, lymph vascular space invasion*.

**Figure 2:**
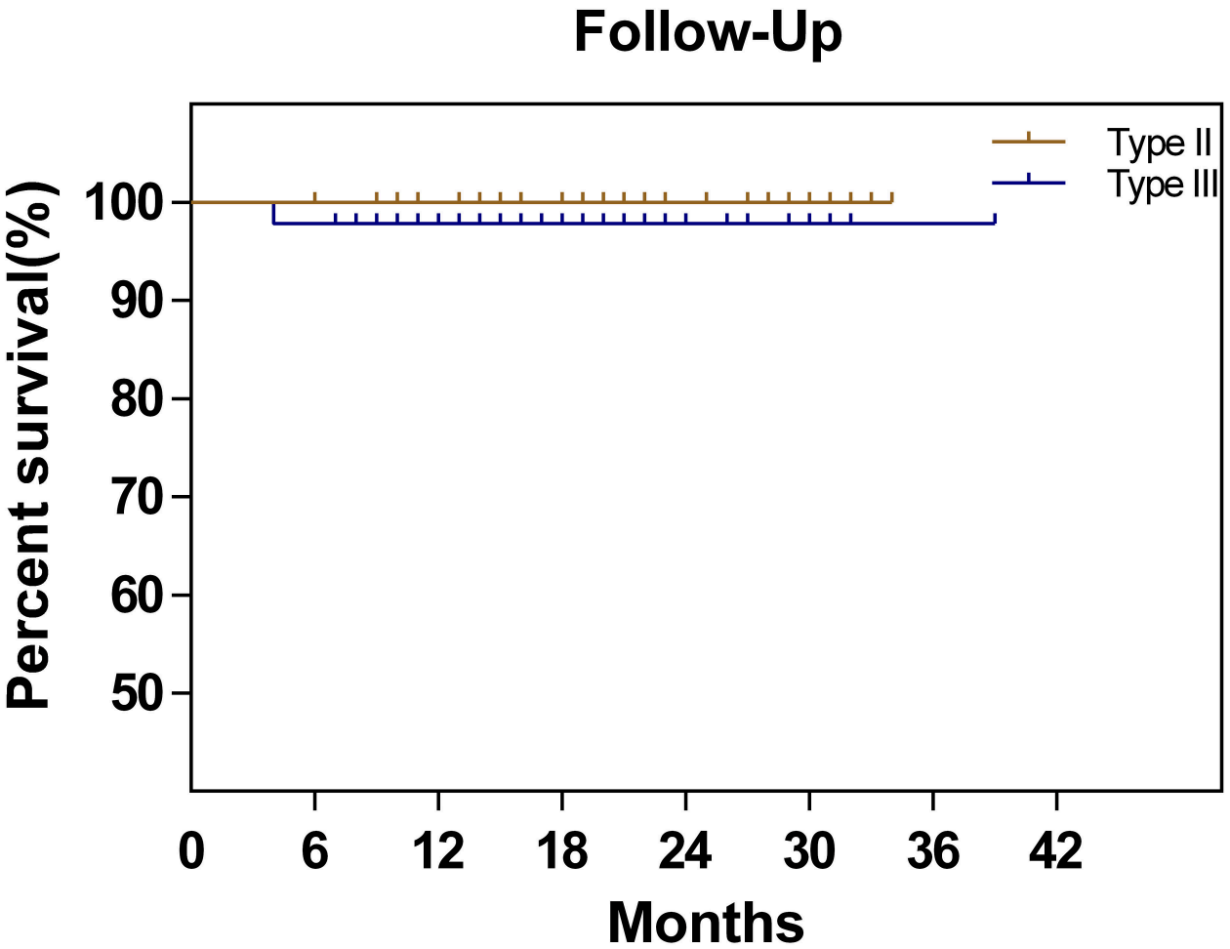
Disease-free survival in patients with cervical cancer treated by type II hysterectomy or type III hysterectomy.

The follow-up was performed until August 2018. The median follow-up time was 28 (6–38) months in the midterm analysis. During the follow-up period, only one DFS event (2.1%), recurrence at 4 months postoperatively, occurred in the type III group, while no DFS events were observed in the type II group. The corresponding 2-year DFS rates were 97.9 and 100%, respectively. The remaining 92 patients all attended a regular follow-up (Figure 2). All patients completed preoperative urodynamic examinations and the QLQ-CX24, FSDS, and FSFI questionnaires. More than 95% of the patients completed urodynamic examinations and questionnaires regarding quality of life at 6 months postoperatively. The percentages of patients who completed the QLQ-CX24, FSDS, and FSFI questionnaires at 12 months postoperatively were 72, 72, and 71%, respectively, and these percentages were 38.7, 35.5, and 33.3 at 24 months postoperatively, respectively (Table 3).

**Table 3:** Patient related outcomes measure completion rate for each surgical group assignment.

**Patient related outcomes measure and assessment time-point**	**Type II cohort (*****N*** = **46)**		**Type III cohort (*****N*** = **47)**		**Total participants of QOL (*****N*** = **93)**	
	***N***	**%**	***N***	**%**	***N***	**%**
**URODYNAMIC STUDY**						
Presurgery	46	100	47	100	93	100
6 months post-surgery	46	100	46	97.9	92	98.9
**EORTC QLQ-CX24**						
Presurgery	46	100	47	100	93	100
6 months post-surgery	46	100	46	97.9	92	98.9
12 months post-surgery	35	76.1	32	68.1	67	72.0
24 months post-surgery	18	39.1	18	38.3	36	38.7
**FSDS**						
Presurgery	46	100	47	100	93	100
6 months post-surgery	46	100	46	97.9	92	98.9
12 months post-surgery	35	76.1	32	68.1	67	72.0
24 months post-surgery	18	39.1	15	31.9	33	35.5
**FSFI**						
Presurgery	46	100	47	100	93	100
6 months post-surgery	46	100	46	97.9	92	98.9
12 months post-surgery	34	73.9	32	68.1	66	71.0
24 months post-surgery	16	34.8	15	31.9	31	33.3

*EORTC QLQ-CX24, european organization for research and treatment of cancer quality of life questionnaire cervical cancer module; FSFD, female sexual distress scale; FSFI, female sexual functioning index; QOL, quality of life*.

The urodynamic detection results are shown in Table 4. All indicators in the preoperative urodynamic examinations were not significantly different between the two groups. At 6 months postoperatively, the maximum cystometric capacity of the patients was decreased in the type III group compared to that in the type II group (322.6 ± 44.7 ml vs. 438.8 ± 33.1 ml, *P* = 0.040). Other indicators were not different between the two groups. However, in a subgroup analysis, the maximum cystometric capacity and the maximum detrusor pressure (Pdet-max) of the patients in the type III group were both significantly decreased compared to those in the type II group (360.4 ± 32.7 ml vs. 446.8 ± 27.9 ml, *P* = 0.048; 24.4 ± 3.0 vs. 34.9 ± 4.1, *P* = 0.045, respectively) at 6 months postoperatively. The maximum flow rate (Qmax) in the patients in the type III group was decreased, although not significantly, compared to that in the type II group (16.2 ± 2.2 vs. 23.5 ± 3.4, *P* > 0.05) (Table 5).

**Table 4:** Comparison of urodynamic study in patients treated with Type II and Type III hysterectomy cohort.

	**Type II cohort**	**Type III cohort**	***P*-value**
Presurgery	*N* = 46	*N* = 47
First sensation cystometric capacity	166.4 ± 11.7	159.3 ± 15.5	NS
Maximum cystometric capacity	396.5 ± 27.4	388.9 ± 16.3	NS
RV	13.4 ± 7.7	14.6 ± 9.6	NS
Qmax	24.7 ± 3.4	27.6 ± 3.1	NS
Pdet-max	37.7 ± 6.9	34.9 ± 5.1	NS
Bladder compliance	65.7 ± 17.6	70.4 ± 26.4	NS
6 months post-surgery	*N* = 46	*N* = 46
First sensation cystometric capacity	183.8 ± 37.0	152.4 ± 26.3	NS
Maximum cystometric capacity	438.8 ± 33.1	322.6 ± 44.7	0.040
RV	11.2 ± 6.9	15.8 ± 8.3	NS
Qmax	20.8 ± 5.2	17.9 ± 3.3	NS
Pdet-max	29.2 ± 8.4	24.6 ± 3.1	NS
Bladder compliance	61.5 ± 17.2	55.8 ± 26.5	NS

*RV, residual urine; Qmax, maximum flow rate; Pdet-max, maximal detrusor pressure; NS, not significant*.

**Table 5:** Subgroup analysis of urodynamic study in patients treated with Type II and Type III hysterectomy cohort without postoperative chemoradiotherapy.

	**Type II cohort**	**Type III cohort**	***P*-value**
Presurgery	*N* = 36	*N* = 35
First sensation cystometric capacity	167.6 ± 11.9	159.2 ± 14.2	NS
Maximum cystometric capacity	396.7 ± 22.7	387.7 ± 17.9	NS
RV	13.8 ± 8.2	14.8 ± 9.1	NS
Qmax	24.5 ± 3.1	24.8 ± 4.2	NS
Pdet-max	36.9 ± 7.7	34.4 ± 4.8	NS
Bladder compliance	68.6 ± 17.4	70.8 ± 24.2	NS
6 months post-surgery	*N* = 36	*N* = 34
First sensation cystometric capacity	171.8 ± 33.0	154.4 ± 25.3	NS
Maximum cystometric capacity	446.8 ± 27.9	360.4 ± 32.7	0.048
RV	12.8 ± 6.1	15.6 ± 7.1	NS
Qmax	23.5 ± 3.4	16.2 ± 2.2	0.079
Pdet-max	34.9 ± 4.1	24.4 ± 3.0	0.045
Bladder compliance	62.6 ± 15.8	54.6 ± 17.5	NS

*RV, residual urine; Qmax, maximum flow rate; Pdet-max, maximal detrusor pressure; NS, not significant*.

The preoperative baseline scores on the QLQ-CX24, FSDS, and FSFI assessments were similar between the two groups (Table 6). At 6 and 12 months postoperatively, the symptom experience scores on the QLQ-CX24 in the type II group were significantly decreased compared to those in the type III group (15.76 ± 2.83 vs. 25.18 ± 3.56, *P* = 0.042; 8.38 ± 2.36 vs. 16.56 ± 3.33, *P* = 0.046, respectively). Within the first 6 months after surgery, 75% of the patients reported having no sexual life, indicating reduced responses to sexual/vaginal functioning, sexual activity, and sexual enjoyment. The differences in symptom experience scores between the two groups were not statistically significant at 24 months (7.24 ± 0.76 vs. 9.85 ± 1.13, *P* = 0.064). The FSDS and FSFI scores in the type II group and type III group were 38.28 ± 4.87 and 22.67 ± 2.37 compared with 37.77 ± 3.39 and 26.53 ± 3.82 at 6 months postoperatively (*P* > 0.05), 36.28 ± 4.27 and 12.84 ± 2.66 compared with 40.11 ± 5.33 and 11.82 ± 2.26 at 12 months postoperatively (*P* > 0.05), and 41.75 ± 6.13 and 14.11 ± 2.05 compared with 44.46 ± 5.06 and 10.88 ± 2.32 at 24 months postoperatively, respectively (*P* > 0.05) (Table 7).

**Table 6:** Mean scores for each patient reported outcomes assessment between Type II and Type III hysterectomy cohort at pre-surgery and 6 months post-surgery.

**Patient reported outcomes measure and assessment point**	**Type II cohort**		**Type III cohort**		***P*-value**
	***N***	**Mean ± SE**	***N***	**Mean ± SE**
**PRE-SURGERY**					
EORTC QLQ-CX24	46		47	
Symptom experience^a^		6.14 ± 0.89		6.26 ± 0.75	NS
Body image^a^		7.22 ± 2.25		7.96 ± 1.77	NS
Sexual/vaginal functioning^a^		5.49 ± 2.88		7.10 ± 2.81	NS
Lymphoedema^a^		6.24 ± 1.97		5.82 ± 2.16	NS
Peripheral neuropathy^a^		8.41 ± 1.56		9.61 ± 2.97	NS
Menopausal symptoms^a^		10.64 ± 2.42		14.05 ± 2.49	NS
Sexual worry^a^		10.46 ± 2.65		12.86 ± 2.18	NS
Sexual activity^b^		9.63 ± 2.77		12.31 ± 2.40	NS
Sexual enjoyment^b^		32.36 ± 6.67		28.31 ± 5.24	NS
FSDS^b^		44.47 ± 6.36		40.86 ± 4.81	NS
FSFI^b^		16.06 ± 2.02		17.87 ± 1.96	NS
Desire		2.32 ± 0.19		2.83 ± 0.33	NS
Arousal		2.14 ± 0.33		2.91 ± 0.48	NS
Lubrication		2.68 ± 0.63		2.50 ± 0.52	NS
Orgasm		2.11 ± 0.27		2.21 ± 0.36	NS
Satisfaction		4.51 ± 0.41		4.66 ± 0.54	NS
Pain		2.25 ± 0.35		2.76 ± 0.47	NS
**6 MONTHS POST-SURGERY**					
EORTC QLQ-CX24	46		46	
Symptom Experience^a^	46	15.76 ± 2.83	46	25.18 ± 3.56	0.042
Body image^a^	46	10.77 ± 3.32	46	16.42 ± 5.02	NS
Sexual/vaginal functioning^a^	13	19.68 ± 4.88	10	26.83 ± 5.69	NS
Lymphoedema^a^	46	24.24 ± 2.12	46	19.45 ± 4.07	NS
Peripheral neuropathy^a^	46	11.36 ± 2.32	46	14.52 ± 2.78	NS
Menopausal symptoms^a^	46	14.53 ± 3.83	46	14.66 ± 3.58	NS
Sexual worry^a^	46	28.81 ± 4.63	46	30.23 ± 5.36	NS
Sexual activity^b^	13	17.67 ± 3.56	10	20.02 ± 4.40	NS
Sexual enjoyment^b^	13	10.21 ± 2.27	10	9.24 ± 2.85	NS
FSDS^b^	46	38.28 ± 4.87	46	37.77 ± 3.39	NS
FSFI^b^	46	22.67 ± 2.37	46	26.53 ± 3.82	NS
Desire	46	2.06 ± 0.33	46	2.85 ± 0.25	NS
Arousal	46	1.93 ± 0.71	46	2.67 ± 0.65	NS
Lubrication	13	3.42 ± 0.78	10	3.97 ± 0.82	NS
Orgasm	13	2.68 ± 0.63	10	2.90 ± 0.64	NS
Satisfaction	13	3.23 ± 0.42	10	3.41 ± 0.58	NS
Pain	13	1.97 ± 0.34	10	2.24 ± 0.66	NS

a *higher scores represent a higher level of symptoms or problems*.

b *higher scores indicate a higher level of functioning and a better quality of life; QOL, quality of life; NS, not significant*.

**Table 7:** Mean scores for each patient reported outcomes assessment between Type II and Type III hysterectomy cohort at 12 and 24 months post-surgery.

**Patient reported outcomes measure and assessment point**	**Type II cohort**		**Type III cohort**		***P*-value**
	***N***	**Mean ± SE**	***N***	**Mean ± SE**
**12 MONTHS POST-SURGERY**					
EORTC QLQ-CX24	35		32	
Symptom experience^a^	35	8.38 ± 2.36	32	16.56 ± 3.33	0.046
Body image^a^	35	7.78 ± 2.48	32	11.90 ± 3.21	NS
Sexual/vaginal functioning^a^	26	24.63 ± 9.44	22	36.17 ± 13.83	NS
Lymphoedema^a^	35	7.76 ± 3.85	32	9.60 ± 6.21	NS
Peripheral neuropathy^a^	35	12.83 ± 3.20	32	10.95 ± 3.35	NS
Menopausal symptoms^a^	35	12.52 ± 4.35	32	12.65 ± 3.26	NS
Sexual worry^a^	35	17.98 ± 2.39	32	19.44 ± 2.28	NS
Sexual activity^b^	26	11.58 ± 3.41	22	7.25 ± 1.99	NS
Sexual enjoyment^b^	26	32.02 ± 5.87	22	21.30 ± 5.54	NS
FSDS^b^	35	36.28 ± 4.27	32	40.11 ± 5.33	NS
FSFI^b^	34	12.84 ± 2.66	32	11.82 ± 2.26	NS
Desire	34	2.03 ± 0.25	32	2.21 ± 0.44	NS
Arousal	34	1.62 ± 0.18	32	1.61 ± 0.31	NS
Lubrication	24	2.46 ± 0.58	21	1.66 ± 0.40	NS
Orgasm	24	1.89 ± 0.42	21	2.01 ± 0.38	NS
Satisfaction	24	2.22 ± 0.31	21	2.13 ± 0.28	NS
Pain	24	1.67 ± 0.42	21	1.96 ± 0.59	NS
**24 MONTHS POST-SURGERY**					
EORTC QLQ-CX24	18		18	
Symptom experience^a^	18	7.24 ± 0.76	18	9.85 ± 1.13	0.064
Body image^a^	18	4.98 ± 1.68	18	6.74 ± 1.87	NS
Sexual/vaginal functioning^a^	12	28.87 ± 8.52	10	31.23 ± 8.86	NS
Lymphoedema^a^	18	3.56 ± 1.61	18	6.81 ± 1.88	NS
Peripheral neuropathy^a^	18	7.94 ± 2.41	18	10.82 ± 3.74	NS
Menopausal symptoms^a^	18	7.66 ± 2.24	18	14.36 ± 2.53	NS
Sexual worry^a^	18	9.48 ± 1.56	18	8.70 ± 1.29	NS
Sexual activity^b^	12	7.10 ± 1.89	10	6.21 ± 2.23	NS
Sexual enjoyment^b^	12	30.04 ± 6.85	10	26.80 ± 5.62	NS
FSDS^b^	18	41.75 ± 6.13	15	44.46 ± 5.06	NS
FSFI^b^	16	14.11 ± 2.05	15	10.88 ± 2.32	NS
Desire	16	2.41 ± 0.64	15	1.86 ± 0.47	NS
Arousal	16	2.29 ± 0.57	15	1.64 ± 0.55	NS
Lubrication	12	2.77 ± 0.44	10	1.89 ± 0.49	NS
Orgasm	12	2.48 ± 0.41	10	2.00 ± 0.52	NS
Satisfaction	12	2.03 ± 0.28	10	2.42 ± 0.36	NS
Pain	12	2.68 ± 0.59	10	2.24 ± 0.73	NS

a *higher scores represent a higher level of symptoms or problems*.

b *higher scores indicate a higher level of functioning and a better quality of life; QOL, quality of life; NS, not significant*.

In a subgroup analysis, the symptom experience scores on the QLQ-CX24 in the type II group were also significantly decreased compared to those in the type III group (16.54 ± 2.73 vs. 23.63 ± 2.62, *P* = 0.036), and no differences in the other dimensions of the QLQ-CX24 were noted at 6 months postoperatively. The FSDS and FSFI scores in the type II group were 40.70 ± 6.87 and 21.67 ± 2.47 compared with 37.64 ± 5.54 and 22.86 ± 3.52 in the type III group, respectively (*P* > 0.05) (Table 8).

**Table 8:** Mean scores between Type II and Type III hysterectomy subgroup without postoperative chemoradiotherapy at Pre-surgery and 12 months post-surgery.

**Patient reported outcomes measure and assessment point**	**Type II cohort**		**Type III cohort**		***P*-value**
	***N***	**Mean ± SE**	***N***	**Mean ± SE**
**PRE-SURGERY**					
EORTC QLQ-CX24	36		35	
Symptom experience^a^		6.14 ± 0.89		6.26 ± 0.77	NS
Body image^a^		7.47 ± 2.24		7.88 ± 1.76	NS
Sexual/vaginal functioning^a^		5.86 ± 2.32		7.71 ± 2.14	NS
Lymphoedema^a^		6.28 ± 1.86		5.72 ± 2.29	NS
Peripheral neuropathy^a^		8.21 ± 1.85		9.77 ± 2.42	NS
Menopausal symptoms^a^		10.54 ± 1.65		13.66 ± 1.79	NS
Sexual worry^a^		9.56 ± 2.44		12.68 ± 2.74	NS
Sexual activity^b^		9.32 ± 2.36		11.24 ± 2.36	NS
Sexual enjoyment^b^		31.36 ± 4.87		28.33 ± 5.54	NS
FSDS^b^		42.74 ± 3.36		44.86 ± 4.81	NS
FSFI^b^		15.44 ± 2.41		17.18 ± 1.86	NS
Desire		2.32 ± 0.33		2.48 ± 0.31	NS
Arousal		2.14 ± 0.36		2.39 ± 0.42	NS
Lubrication		2.38 ± 0.46		2.68 ± 0.38	NS
Orgasm		1.98 ± 0.34		2.40 ± 0.37	NS
Satisfaction		4.18 ± 0.56		4.61 ± 0.45	NS
Pain		2.44 ± 0.48		2.62 ± 0.43	NS
**6 MONTHS POST-SURGERY**					
EORTC QLQ-CX24	36		34	
Symptom experience^a^	36	16.54 ± 2.73	34	23.63 ± 2.62	0.036
Body image^a^	36	8.68 ± 2.87	34	15.56 ± 4.29	NS
Sexual/vaginal functioning^a^	12	18.82 ± 4.29	8	23.63 ± 5.41	NS
Lymphoedema^a^	36	26.95 ± 1.86	34	20.65 ± 3.99	NS
Peripheral neuropathy^a^	36	9.64 ± 1.42	34	13.82 ± 2.66	NS
Menopausal symptoms^a^	36	11.43 ± 2.71	34	12.71 ± 1.82	NS
Sexual worry^a^	36	26.43 ± 3.44	34	27.88 ± 2.39	NS
Sexual activity^b^	12	15.24 ± 2.78	8	19.82 ± 3.47	NS
Sexual enjoyment^b^	12	12.46 ± 2.64	8	9.85 ± 2.74	NS
FSDS^b^	36	40.70 ± 6.87	34	37.64 ± 5.54	NS
FSFI^b^	36	21.67 ± 2.47	34	22.86 ± 3.52	NS
Desire	36	1.84 ± 0.37	34	2.47 ± 0.36	NS
Arousal	36	1.74 ± 0.43	34	2.57 ± 0.64	NS
Lubrication	12	2.52 ± 0.64	8	3.05 ± 0.81	NS
Orgasm	12	2.47 ± 0.62	8	2.61 ± 0.47	NS
Satisfaction	12	3.67 ± 0.34	8	3.83 ± 0.55	NS
Pain	12	2.38 ± 0.53	8	2.70 ± 0.42	NS

a *higher scores represent a higher level of symptoms or problems*.

b *higher scores indicate a higher level of functioning and a better quality of life; QOL, quality of life; NS, not significant*.

At 12 months postoperatively, the symptom experience scores in the type II and type III groups were 7.69 ± 1.78 and 13.62 ± 2.23, respectively (*P* = 0.045). The sexual/vaginal functioning scores on the QLQ-CX24 in the type II group were significantly lower than those in the type III group (20.85 ± 3.38 vs. 33.74 ± 4.79, *P* = 0.036), while scores on the other dimensions of the QLQ-CX24 were not different between the two groups. The total FSDS and FSFI scores in the type II group were 41.43 ± 4.18 and 13.47 ± 1.16 compared with 40.20 ± 6.84 and 13.86 ± 1.44 in the type III group, respectively (*P* > 0.05). However, the lubrication score on the FSFI scale in the type II group was significantly higher than that in the type III group (2.79 ± 0.29 vs. 1.42 ± 0.52, *P* = 0.032) (Table 9).

**Table 9:** Mean scores between Type II and Type III hysterectomy subgroup without postoperative chemoradiotherapy at 12 and 24 months post-surgery.

**Patient reported outcomes measure and assessment point**	**Type II cohort**		**Type III cohort**		***P* value**
	***N***	**Mean ± SE**	***N***	**Mean ± SE**
**12 MONTHS POST-SURGERY**					
EORTC QLQ-CX24	28		24	
Symptom experience^a^	28	7.69 ± 1.78	24	13.62 ± 2.23	0.045
Body Image^a^	28	6.58 ± 2.74	24	9.96 ± 3.30	NS
Sexual/vaginal functioning^a^	18	20.85 ± 3.38	19	33.74 ± 4.79	0.036
Lymphoedema^a^	28	6.65 ± 3.36	24	9.65 ± 2.43	NS
Peripheral neuropathy^a^	28	11.54 ± 3.60	24	8.33 ± 3.55	NS
Menopausal symptoms^a^	28	12.67 ± 3.13	24	10.81 ± 3.22	NS
Sexual worry^a^	28	16.74 ± 3.89	24	18.11 ± 4.21	NS
Sexual activity^b^	19	12.62 ± 4.23	24	8.86 ± 2.97	NS
Sexual enjoyment^b^	19	33.38 ± 6.48	24	23.88 ± 5.87	NS
FSDS^b^	27	41.43 ± 4.18	24	40.20 ± 6.84	NS
FSFI^b^	23	13.47 ± 1.16	24	13.86 ± 1.44	NS
Desire	23	2.04 ± 0.35	24	2.18 ± 0.32	NS
Arousal	23	1.87 ± 0.43	24	1.69 ± 0.41	NS
Lubrication	18	2.79 ± 0.29	19	1.42 ± 0.52	0.032
Orgasm	18	2.38 ± 0.57	19	2.16 ± 0.61	NS
Satisfaction	18	2.78 ± 0.50	19	2.24 ± 0.42	NS
Pain	18	1.68 ± 0.47	19	2.03 ± 0.53	NS
**24 MONTHS POST-SURGERY**					
EORTC QLQ-CX24	14		15	
Symptom experience^a^	14	6.77 ± 0.94	15	6.45 ± 1.15	NS
Body image^a^	14	4.89 ± 2.53	15	6.01 ± 2.17	NS
Sexual/vaginal functioning^a^	11	18.45 ± 3.10	9	28.98 ± 3.61	0.039
Lymphoedema^a^	14	3.33 ± 1.04	15	6.17 ± 1.36	NS
Peripheral neuropathy^a^	14	7.31 ± 1.82	15	10.32 ± 3.08	NS
Menopausal symptoms^a^	14	7.25 ± 2.56	15	12.31 ± 3.13	NS
Sexual worry^a^	14	9.38 ± 3.25	15	8.75 ± 2.34	NS
Sexual activity^b^	11	7.68 ± 2.45	9	6.37 ± 1.98	NS
Sexual enjoyment^b^	11	30.15 ± 5.15	9	27.80 ± 4.74	NS
FSDS^b^	14	43.83 ± 5.75	14	44.47 ± 4.89	NS
FSFI^b^	14	14.74 ± 2.33	14	11.79 ± 2.93	NS
Desire	14	2.47 ± 0.43	14	1.98 ± 0.32	NS
Arousal	14	2.54 ± 0.49	14	1.78 ± 0.61	NS
Lubrication	11	2.82 ± 0.28	9	1.88 ± 0.34	0.047
Orgasm	11	2.69 ± 0.37	9	2.12 ± 0.28	NS
Satisfaction	11	2.21 ± 0.13	9	2.55 ± 0.24	NS
Pain	11	2.47 ± 0.48	9	2.25 ± 0.40	NS

a *higher scores represent a higher level of symptoms or problems*.

b *higher scores indicate a higher level of functioning and a better quality of life; QOL, quality of life; NS, not significant*.

At 24 months postoperatively, the sexual/vaginal functioning score on the QLQ-CX24 in the type II group was significantly lower than that in the type III group (18.45 ± 3.10 vs. 28.98 ± 3.61, *P* = 0.039). The FSDS score in the type II group was 43.83 ± 5.75, which was not different from the score of 44.47 ± 4.89 in the type III group (*P* > 0.05). The total FSFI score was not different between the two groups (14.74 ± 2.33 vs. 11.79 ± 2.93, *P* > 0.05), but the lubrication score on the FSFI questionnaire in the type II group was higher than that in the type III group (2.82 ± 0.28 vs. 1.88 ± 0.34, *P* = 0.047). However, because fewer patients completed the questionnaire in this period, the validity should be further analyzed (Table 9).

**Figure 3:**
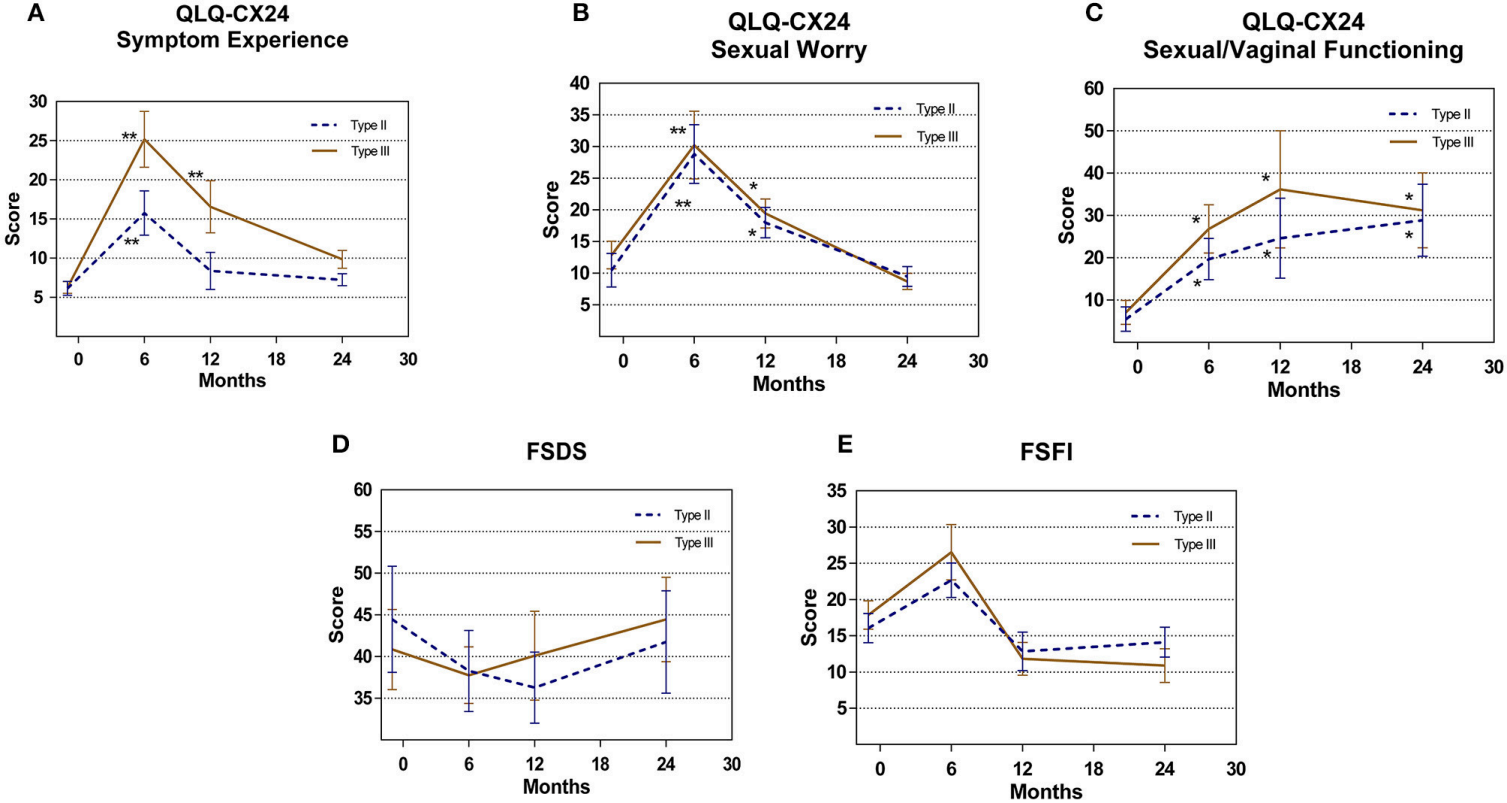
Patient related outcomes measure **(A)** QLQ-CX24 Symptom Experience **(B)** QLQ-CX24 Sexual Worry **(C)** QLQ-CX24 Sexual/Vaginal Functioning **(D)** FSDS **(E)** FSFI comparison for type II vs. type III group. *P < 0.05, **P < 0.01.

For all patients, the mean sexual apprehension scores on the QLQ-CX24 at 6 and 12 months postoperatively were 29.52 ± 4.88 and 18.74 ± 2.51, respectively, a significant increase compared with the score of 11.66 ± 2.27 before surgery (*P* = 0.001, *P* = 0.038, respectively). However, the score recovered to 9.09 ± 1.43 at 24 months postoperatively. The postoperative sexual/vaginal functioning scores on the QLQ-CX24 in both groups increased after surgery (*P* < 0.05). The overall FSDS and FSFI scores were not different pre- and post-surgery (Figure 3).

## Discussion

For early-stage cervical cancer patients with FIGO IA2-IB1 stage disease, the NCCN currently recommends radical hysterectomy combined with systematic lymphadenectomy. However, the surgical range is too large, which severely affects the quality of life of patients (18, 19).

Van Meurs et al. noted that no parametrial infiltration occurred in 1,063 patients with stage IA2 cervical cancer. However, 4.8% of the patients had LN metastasis and the recurrence rate was 3.6% (19). Covens et al. retrospectively analyzed 842 patients with stage IB1 cervical cancer and noted that the risk of parametrial infiltration in low-risk patients (tumor diameter < 2 cm, interstitial infiltration depth < 10 mm, and negative pelvic LNs) was 0.6% (20). Our previous multicenter study indicated that among 1,123 patients with stage IA2 and IB1 (maximum tumor diameter < 2 cm) cervical cancer, the parametrial and vaginal involvement rates were both 0.2% (2/1,123), the uterine isthmus involvement rate was 1.8% (20/1,123), and the pelvic LN metastasis rate was 6.1% (69/1,123) (21, 22). Moreover, the JCOG0806-A study retrospectively analyzed patients with stage IB1 cervical cancer and reported that the 5-year overall survival rate was 95.8% and that pathological parametrial involvement was observed in only 1.9% of low-risk patients (2). To evaluate the security of modified radical hysterectomy, Xie analyzed 86 patients with early-stage cervical cancer (IB1-IAl) who underwent modified vaginal radical hysterectomy and system lymphadenectomy and reported that the recurrence rate and overall survival rate were 3.57 and 97.62% within 46 months, respectively (23). In 2013, the Gynecologic Cancer Study Group of the Japan Clinical Oncology Group (JCOG) initiated a multicenter study to evaluate the efficacy of Piver II hysterectomy in FIGO Stage IB1 low-risk cervical cancer patients (JCOG1101), and we look forward to their reports (24).

The present study systematically evaluated the safety and quality of life associated with type II and type III hysterectomy with systematic lymphadenectomy in the treatment of stage IA2-IB1 cervical cancer patients with a maximum tumor diameter < 2 cm. The results showed that the surgical time and intraoperative blood loss in the type II group was significantly lower than those in the type III group, mainly because of the reduced surgical range and minimal blood vessel injuries (25). The present study showed that the effects of these two surgical methods on postoperative aerofluxus and defecation functions were not different. However, different effects on urinary function were found. The number of patients with urinary retention within 14 days postoperatively was lower in the type II group than that in the type III group, which may be associated with bladder retroflexion caused by pelvic nerve injury and the larger resection range of uterine, vaginal, and parametrial tissues with type III hysterectomy (26). The positive rate of LVSI in low-risk, early-stage cervical cancer has been reported to be 10.3–45%. The positive rate of LVSI was 23.7% in the present study, which was the major risk factor for postoperative chemoradiotherapy (26, 27).

To effectively evaluate changes in bladder function and quality of life among patients, we excluded possible confounding factors (patients aged >60 years and those with other diseases) to control biases. Moreover, postoperative chemoradiotherapy was also identified as a confounding factor (28); therefore, we performed subgroup analyses in the two groups without postoperative chemoradiotherapy. The maximum cystometric capacity and Pdet-max of the patients in the type II group were significantly higher at 6 months postoperatively, suggesting that the detrusor function of the patients in the type III group had different degrees of injury, which caused a reduction in the Qmax and affected urinary function. Chen et al. reported that the number of patients with detrusor instability after surgery significantly increased, which may be associated with local sympathetic and parasympathetic nerve injury (29). The subsequent study by Todo on nerve-sparing surgery also confirmed this point (30).

Jensen et al. reported that the lack of sexual interest among cervical cancer patients after radical hysterectomy persisted for 2 years (31). The present study showed that most patients had lower sexual activity overall after surgery, which may be due to sexual apprehension within 2 years postoperatively. Therefore, we suggest that psychological factors are important and affect postoperative quality of life and sexual function. The sexual enjoyment and desire scores were not significantly different in our study, which may be related to the conservative nature of Asian females in response of sexual function-related questions (15, 17).

Furthermore, better sexual/vaginal functioning and lubrication were noted among the patients in the type II group, and symptoms in the type II group were significantly attenuated according to the subgroup analysis, which may be due to resection of less parametrial and vaginal tissues and decreased rates of pelvic nerve injury, scar fibrosis, and vaginal blood circulation disorders in the type II group (32, 33). The symptom experience scores in the type III group were significantly increased compared to those in the type II group at 6 and 12 months postoperatively, and these scores recovered at 24 months postoperatively, possibly due to recovery from surgical trauma and postoperative complications (34, 35). In addition to the differences in other indicators in all patients before and after surgery, we considered that this finding may have been observed because other indicators, such as sexual apprehension, sexual activity, and sexual enjoyment, were mainly associated with the psychological and neuroendocrine factors of malignant oncology and related surgery, which are rarely influenced by the type of radical hysterectomy (31). Moreover, the ovaries are not routinely removed in both types of surgical procedure, and menopausal symptoms were therefore not significantly different because such symptoms are mainly regulated by ovarian hormones, which can be affected by different surgery types. Similarly, the postoperative lymphedema score was significantly higher than the preoperative score in all patients, which was mainly related to pelvic lymphadenectomy (36). Therefore, no differences in surgical types were observed. The scores for sexual activity, sexual enjoyment, orgasm, and satisfaction dimensions were not different between the two groups at each time point postoperatively, which is inconsistent with the conclusion reported by Francesco Plotti (37). It should be noted that we performed an overall analysis of the low-risk, early-stage cervical cancer population rather than patients who were young and sexually active. In addition, baseline scoring of the preoperative quality of life and sexual life status of all patients was performed to control for differences between the groups before surgery. Moreover, the characteristics (including ethnic factors, educational background, physical factors, and psychological factors) of patients may differ across studies. The enrolled patients were all Chinese in our study, which may also reflect a distinguishing characteristic of our study population. The 2-year survival rates of the type II and type III groups were 100 and 97.6%, respectively, which is consistent with the rates reported in the literature (38, 39). The security of type II hysterectomy combined with systematic lymphadenectomy in the treatment of low-risk, early-stage cervical cancer patients was not worse than that for the type III hysterectomy patients within 2 years after surgery. Certainly, more patients will be enrolled in our next study.

## Conclusion

Overall, based on the midterm analysis, type II hysterectomy effectively decreased the surgical time and intraoperative blood loss, reduced bladder complications, increased patients' quality of life, and showed favorable security in low-risk, early-stage cervical cancer within 2 years after surgery. However, we should also focus more on the postoperative apprehension of patients.

## Ethics statement

Ethical approval: All procedures in studies involving human participants were performed in accordance with the ethical standards of the institutional and national research committee and with the 1964 Declaration of Helsinki and its later amendments (the name and affiliation of the ethics committee that approved this study: The Institutional Ethics Committee of Peking Union Medical College Hospital, CAMS Chinese Academy of Medical Sciences, No. S-785 2015. Each institution received Institutional Review Board (IRB) approval).

Written informed consent was obtained from all participants included in the study.

## Author contributions

DC and KS: protocol/project development and clinical data provider; HS: data collection, data analysis, manuscript writing, and the follow-up; JY, YX, FF, LW, ZZ, BL, and LS: clinical data providers.

### Conflict of interest statement

The authors declare that the research was conducted in the absence of any commercial or financial relationships that could be construed as a potential conflict of interest.

## Supplementary material

Supplementary Figure 1 The photograph of specimens for quality assurance of surgery. **(A)** Piver type II hysterectomy. **(B)** Piver type III hysterectomy.
