# Supplementary figures and images for: Piver Type II vs. Type III Hysterectomy in the Treatment of Early-Stage Cervical Cancer: Midterm Follow-up Results of a Randomized Controlled Trial
Source: Front Oncol. 2018 Nov 28;8:568. doi: 10.3389/fonc.2018.00568 (PMC6280766; doi:10.3389/fonc.2018.00568)

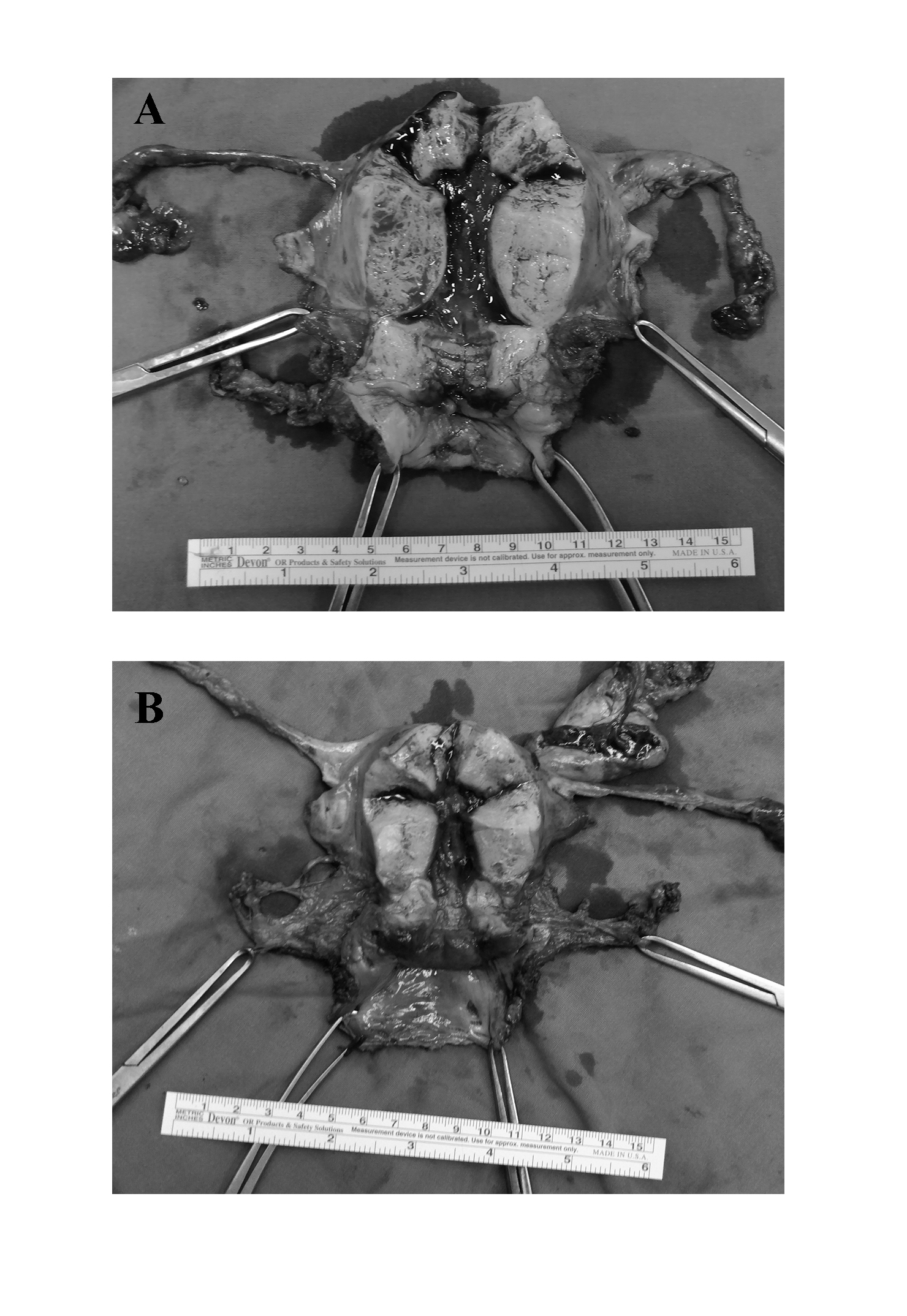

Supplement: Supplementary Figure 1 [file Image_1.JPEG]
